# Red blood cell distribution width-to-albumin ratio and its association with age-related macular degeneration: a population-based cross-sectional study

**DOI:** 10.3389/fmed.2025.1510756

**Published:** 2025-04-15

**Authors:** Ning An, Bing Zeng, Ziling Liu, Chuanhe Zhang, Hongxia Liao, Dongcheng Liu, Bo Qin

**Affiliations:** ^1^Shenzhen Aier Eye Hospital, Aier Eye Hospital, Jinan University, Shenzhen, China; ^2^Shenzhen Aier Ophthalmic Technology Institute, Shenzhen, China; ^3^Aier School of Ophthalmology, Central South University, Changsha, China

**Keywords:** age-related macular degeneration, NHANES, Red blood cell distribution width, albumin, ratio

## Abstract

**Background:**

Age-related macular degeneration (AMD) is a leading cause of central vision impairment in middle-aged and older individuals. There is substantial evidence that AMD is associated with inflammation. The study aimed to investigate the association between the inflammatory marker, red blood cell distribution width/albumin ratio (RAR), and AMD.

**Methods:**

Our study included 5,370 participants aged 40 years and older, using NHANES data from 2005 to 2008. Multivariable logistic regression analysis was conducted to examine the relationship between RAR and AMD in the study. Smooth curves and the piecewise linear regression model were used to determine whether the correlation was linear or non-linear. Additionally, subgroup analysis and interaction testing were performed.

**Results:**

We found a positive linear correlation between RAR and AMD, even after adjusting for covariates. Each unit increase in RAR corresponded to a 30% increase in the odds of AMD prevalence (OR = 1.3; 95% CI, 1.0–1.6). The odds of AMD prevalence were 1.7 times greater in the highest quintile (Q5) group than in the lowest quintile (Q1) group (OR = 1.7; 95% CI, 1.2–2.5). Higher RAR values, compared to lower values, were significantly associated with increased odds of AMD prevalence (*p* trend < 0.05). Subgroup analyses and interaction tests confirmed the stability of the findings.

**Conclusion:**

This study found that there is a positive linear correlation between RAR and the odds of AMD prevalence in United States adults. Further research is necessary to clarify the specific physiological mechanisms underlying the relationship between RAR and AMD.

## 1 Introduction

Age-related macular degeneration (AMD) is a progressive degenerative disease that primarily affects the vision of middle-aged and older people, and it has the potential to cause permanent damage to a patient’s central vision, which can significantly impact their physical and mental wellbeing (1). As the global population ages, AMD is likely to become a significant economic and public health burden on society (2). AMD is classified into several stages based on pathological changes: early AMD, intermediate AMD, and late AMD. Late AMD encompasses two distinct forms: geographic atrophy (dry AMD), which is characterized by confluent atrophy of photoreceptors and the retinal pigment epithelium (RPE), and neovascular AMD (wet AMD), which is marked by neovascular leakage that leads to the accumulation of intraretinal or subretinal fluid, as well as hemorrhages and fibrosis (3). Inflammation drives AMD progression through oxidative stress, complement activation, and pro-inflammatory cytokines. In dry AMD, inflammation accelerates RPE and photoreceptor degeneration, while in wet AMD, it promotes choroidal neovascularization (CNV) via Vascular Endothelial Growth Factor (VEGF) signaling. RPE–immune cell interactions sustain this inflammatory cycle, exacerbating disease progression (4, 5). An increasing body of evidence has substantiated the correlation between systemic inflammation and AMD (6–9). In a case–control study conducted by Ojaghi et al. in 2024, which involved 204 participants, blood samples were collected to measure inflammatory factors, including lymphocytes, monocytes, neutrophils, the neutrophil-to-lymphocyte ratio (NLR), and C-reactive protein (CRP). The study revealed a significant association between the number of neutrophils in peripheral blood and the severity of AMD (9). Numerous other studies have also demonstrated the association between various inflammatory biomarkers and AMD (10–12). Furthermore, a 2023 retrospective study collected data on the Aggregate Index of Systemic Inflammation (AISI) and the Systemic Inflammation Response Index (SIRI) from 90 patients with dry AMD and 270 age- and sex-matched cataract patients. The results revealed no significant differences in AISI and SIRI between the case and control groups. The authors suggested that further exploration of other routine blood biomarkers is necessary for the identification and prevention of AMD (13).

Red blood cell distribution width-to-albumin ratio (RAR) is an innovative biomarker that integrates inflammation and nutritional status. Specifically, red blood cell distribution width (RDW) is a biomarker that indicates the variability in red blood cell volume (14). Numerous studies have indicated that elevated RDW is associated with the occurrence of retinal vascular occlusive diseases, such as retinal artery occlusion (RAO) and retinal vein occlusion (RVO), which are generally considered to be linked to inflammation, oxidative stress, and endothelial dysfunction (15–18). Serum albumin is a significant biomarker for inflammatory response and nutritional status (19, 20). Low serum albumin levels are linked to a poor prognosis in disease outcomes (21, 22). Studies have demonstrated that RAR, which combines RDW and serum albumin, is significantly associated with mortality in critically ill patients with rheumatic diseases (23), sepsis (24), and diabetic foot ulcers (25). Furthermore, RAR plays a crucial role in risk stratification and long-term prognosis prediction for patients with non-ischemic heart failure (26) and intracerebral hemorrhage (27).

Despite the established role of systemic inflammation in AMD, the potential connection between RAR and AMD remains unexplored. It is necessary to investigate the potential connection between RAR and AMD, as this may enhance our understanding of the relationship between systemic inflammation levels and the prevalence of AMD. Therefore, the objective of our study was to explore the relationship between the novel inflammatory marker RAR and AMD using data from the National Health and Nutrition Examination Survey (NHANES).

## 2 Methods

### 2.1 Ethical Approval

All participants signed an informed consent form. The National Center for Health Statistics (NCHS) Research Ethics Review Board (ERB) approved the protocol for the NHANES. According to the website https://www.cdc.gov/nchs/nhanes/irba98.htm, the NCHS Institutional Review Board/Ethics Review Board (IRB/ERB) protocol number for the 2005–2008 NHANES was “#2005–06”.

### 2.2 Study participants

**Figure 1:**
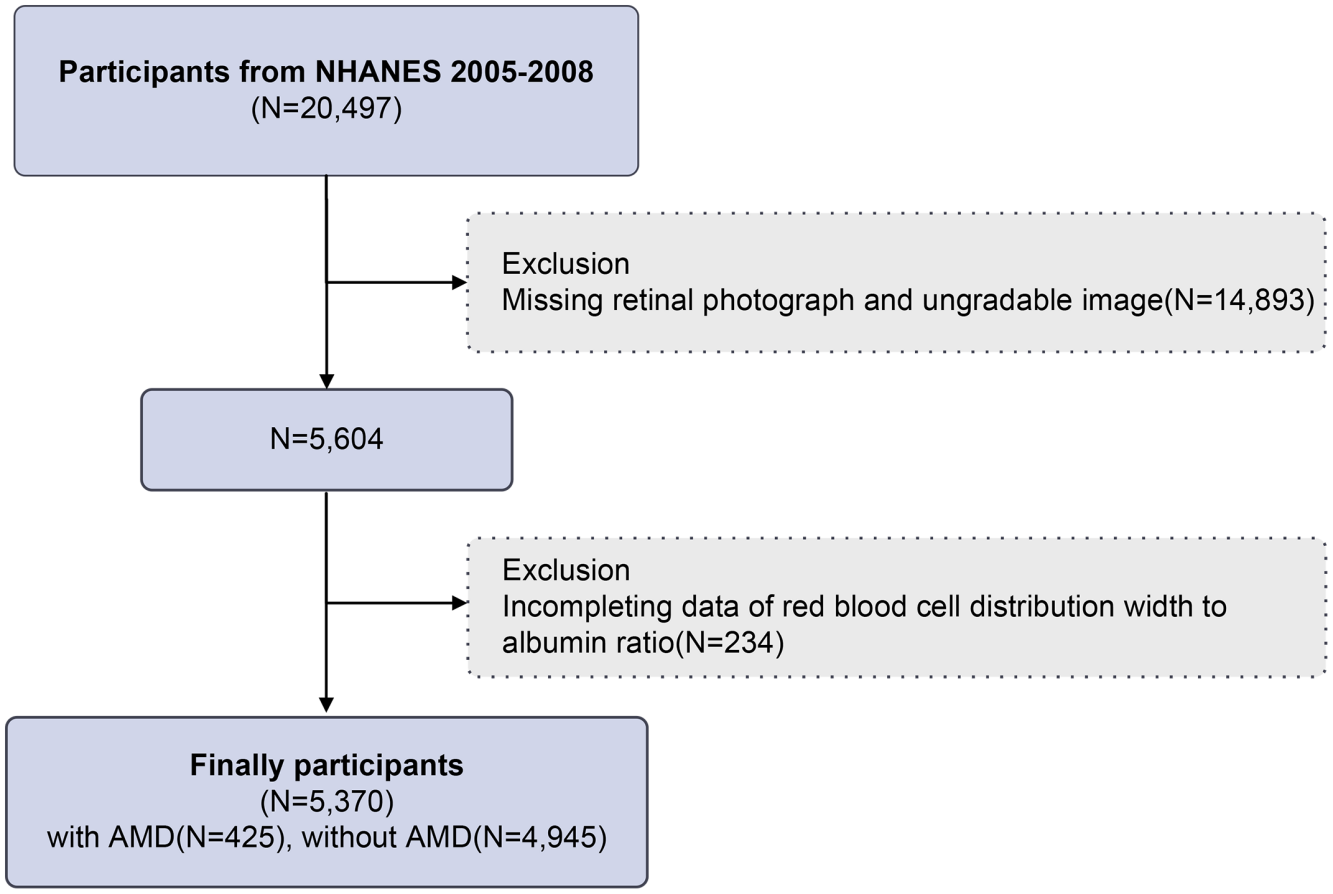
Flowchart of participant selection.

The NHANES database is a nationwide, biennial, cross-sectional research study that collects health-related data from randomly chosen participants to evaluate the health status of the US population. Our research’s data includes two cycles from 2005 to 2008 (2005–2006 and 2007–2008). We excluded 14,893 individuals with missing or ungradable retinal images and 234 people with incomplete RAR data from 20,497 participants; 5,370 participants were eventually included in our research. The screening process is shown in Figure 1.

### 2.3 Exposure and outcomes

All participants’ blood specimens were collected early in the morning of the following day after a 9-h fasting period, and blood cell counts were determined using the Beckman Coulter method. The bichromatic digital endpoint method was used to measure albumin concentration. Using laboratory data from the NHANES, we calculated the ratio of RDW to albumin.

Our study included data from the 2005–2008 NHANES, in which investigators captured two fundus photographs of each eye of individuals aged 40 years and older. The Canon Formula Ophthalmic Imaging equipment and digital cameras were used to capture the retina images (Canon CR6-45NM and EOS 10D). These photographs were systematically evaluated using the Wisconsin Age-Related Maculopathy Grading System by two experienced personnel; if there was any disagreements, a more experienced expert would evaluate them. The fundus photographs were classified into three categories: without AMD, early AMD, and late AMD. Early AMD is defined as the presence or absence of Drusen and/or pigmentary abnormalities; late AMD is characterized by exudative arm signs and/or geographic atrophy (28).

### 2.4 Covariates

Questionnaires and examination data provided substantial information on covariates, including gender, age, ethnicity, general health condition, smoking status: never (<100 cigarettes), former (≥100 cigarettes but quit), and current (≥100 cigarettes and still smoking); alcohol consumption: none, moderate (1 drink per day for women, 1–2 drinks per day for men), heavy (2–3 drinks per day for women, 3–4 drinks per day for men, or ≥4 drinks per day for women, and ≥5 drinks per day for men); and body mass index (BMI) (<18.5, 18.5–25.0, 25.0–30.0, ≥30.0 kg/m2). Diabetes is defined as a history of diabetes or the use of hypoglycemic drugs or insulin, or at least one of the following abnormalities: glycohemoglobin HbA1c (%) > 6.5, fasting glucose (mmol/L) ≥ 7.0, random blood glucose (mmol/L) ≥ 11.1, or 2 h OGTT blood glucose (mmol/L) ≥ 11.1. Hypertension was defined as the average of three measurements of systolic blood pressure ≥140 mmHg and/or diastolic blood pressure ≥90 mmHg, a history of hypertension, or the use of antihypertensive medication. Hyperlipidemia is defined as having a history of hyperlipidemia or taking antihyperlipidemic medications, with total cholesterol ≥5.2 mmol/L, triglyceride levels ≥1.7 mmol/L, LDL-C levels ≥3.4 mmol/L, and HDL-C < 1.0 mmol/L (with abnormalities in at least one of the four laboratory results). Blood test results, including counts of white blood cells (WBC), lymphocytes (LYMP), monocytes (MO), red blood cells (RBC), and platelets (PLT), were also included as covariates.

### 2.5 Statistical analysis

The participants in this study were classified into five groups according to the quintiles of RAR. Continuous and categorical variables were expressed as mean ± SD and percentages (95% confidence intervals, 95% CI), respectively. Weighted linear regression analysis or chi-square tests were used to assess the differences between the groups. Three models of multivariate logistic regression were used to investigate the relationship between RAR and AMD. Model 1 did not adjust for any variables. Model 2 was adjusted for age, gender, and ethnicity. Model 3 was further adjusted for the following variables: smoking status, alcohol consumption, BMI, general health condition, diabetes, hypertension, hyperlipidemia, and counts of WBC, LYMP, MO, RBC, and PLT. Smooth curves and the piecewise linear regression model were used to confirm whether the correlation was linear or non-linear. Subgroup analyses and interaction testing were conducted to examine the consistency of this relationship under various stratification settings. All statistical analyses were performed using EmpowerStats (version 4.2) and R software (version 4.2.0), with a significance level of *p* < 0.05.

## 3 Results

### 3.1 Characteristics of participants

The comprehensive characterization of the 5,370 eligible individuals based on quintiles of RAR is shown in Table 1. The average age of individuals was 56.4 years. Among the participants, 52.4% were women and 47.6% were men. Additionally, AMD was present in 6.6% of the participants. Except for lymphocyte count and smoking status (*p* > 0.05), the majority of the results revealed statistically significant variations across quintiles of RAR.

**Table 1:** Characteristics of participants (*n* = 5,370) based on quintiles of RAR.

	Total	Q1	Q2	Q3	Q4	Q5	*p*-value
	*N* = 5,370	(2.3–2.8)	(2.8–3.0)	(3.0–3.1)	(3.1–3.4)	(3.4–10.2)	
		*N* = 1,067	*N* = 1,078	*N* = 1,038	*N* = 1,104	*N* = 1,083	
Age, year	56.4 ± 11.7	53.6 ± 10.0	55.5 ± 11.0	56.8 ± 11.7	58.7 ± 12.3	59.0 ± 13.4	<0.0001
Gender (%)							<0.0001
Male	47.6	59.3	53	46.6	36.5	35	
Female	52.4	40.7	47	53.5	63.5	65	
Ethnicity (%)							<0.0001
Mexican American	5.5	5.1	5.9	5.7	5.5	5.1	
Other Hispanic	3.2	3.5	3	3.1	2.9	3.5	
Non-Hispanic white people	77.7	82.1	80.9	78.7	77.1	65	
Non-Hispanic black people	9	3.1	5.4	8.2	11.3	22.4	
Other	4.7	6.2	4.8	4.3	3.3	4.1	
Smoking status (%)							0.1483
Never	48.7	50.4	49.5	49	48.4	44.7	
Former	30.8	32.1	29.1	30.5	30	32.5	
Current	20.5	17.6	21.4	20.4	21.5	22.9	
Unknown	§	§	§	§	§	§	
Alcohol consumption (%)							<0.0001
None	11.2	7	10.4	10.6	15.3	14.8	
Moderate	35.1	40.5	36.7	35.3	31.6	27.6	
Heavy	35.7	39.2	37	35.5	33.4	31	
Unknown	18.1	13.4	15.9	18.7	19.8	26.6	
BMI (%)							<0.0001
Underweight	1.2	1.8	0.8	0.8	1.1	1.5	
Normal	26.5	34.5	28.7	26.7	20.3	17.1	
Over weight	35.4	41.2	39.3	33.4	30.9	28	
Obese	36.3	22.4	31	38.2	47.3	51.8	
Unknown	0.6	0.1	0.4	0.9	0.4	1.6	
General health condition (%)							<0.0001
Excellent, very good, or good	18.1	10.1	13.2	15.6	25	33.2	
Fair or poor	80.2	87.6	84.8	83.2	73.6	65.6	
Unknown	1.7	2.4	2	1.2	1.5	1.2	
Diabetes (%)							<0.0001
No	83.7	90.4	87.7	84.7	77.7	72.8	
Yes	16.2	9.6	12.2	15.3	22.2	26.8	
Unknown	0.1	§	0.1	0	0.1	0.4	
Hypertension (%)							<0.0001
No	41.5	45.5	46.5	39.7	38.6	33.1	
Yes	57.9	54	52.6	59.6	61.1	65.9	
Unknown	0.7	0.5	0.9	0.7	0.3	1	
Hyperlipidemia (%)							0.0005
No	19.7	17.9	18.3	17.8	20.7	25.7	
Yes	77.4	78.7	78.8	79.3	77	71.1	
Unknown	3	3.4	2.9	2.9	2.4	3.3	
WBC, 1000cells/ul	7.3 ± 2.3	7.0 ± 1.9	7.2 ± 2.0	7.3 ± 2.7	7.5 ± 2.5	7.6 ± 2.7	<0.0001
LYMP, 1000cells/ul	2.1 ± 1.2	2.1 ± 0.6	2.1 ± 0.7	2.2 ± 1.8	2.2 ± 1.3	2.1 ± 1.6	0.1315
MO, 1000cells/ul	0.6 ± 0.2	0.6 ± 0.2	0.6 ± 0.2	0.6 ± 0.2	0.6 ± 0.2	0.6 ± 0.2	0.0002
RBC, 1000cells/ul	4.7 ± 0.5	4.8 ± 0.4	4.7 ± 0.5	4.7 ± 0.4	4.7 ± 0.5	4.6 ± 0.6	<0.0001
PLT, 1000cells/ul	271.8 ± 69.4	264.8 ± 57.0	268.1 ± 64.5	271.5 ± 67.3	272.6 ± 72.0	287.9 ± 89.0	<0.0001
AMD (%)							<0.0001
No	93.4	95.9	94.7	92.9	91.1	90.5	
Yes	6.6	4.1	5.3	7.1	8.9	9.5	

Mean ± SD was for quantitative variables, % was for qualitative variables; *p*-values are weighted linear regression analysis and chi-square testing; BMI, body mass index; WBC, white blood cell; LYMP, lymphocyte; MO, monocyte; RBC, red blood cell; PLT, platelet; AMD, age-related macular degeneration; RAR, red blood cell distribution width-to-albumin ratio; §: A small sample size resulted in the data not being visible.

Furthermore, individuals with higher levels of RAR were more likely to be older, female, non-Hispanic white, heavy drinkers, and have higher BMI. Notably, compared to individuals without AMD, those with AMD had higher RDW and RAR levels and lower albumin levels, as shown in Tables 1, 2 (all *p*-values < 0.05). Further dividing participants with AMD into early AMD (*N* = 371) and late AMD (*N* = 54), we found that as RAR levels increased, the number of participants with early and late AMD gradually increased, while the number of participants without AMD decreased, based on RAR quintile grouping, as shown in Supplementary Table 1.

**Table 2:** The level of RDW and albumin in the patients with AMD or without AMD.

	Without AMD N = 4,945	With AMD N = 425	*p*-value
RDW	12.8 ± 1.2	13.1 ± 1.2	<0.001
Albumin (g/dl)	4.2 ± 0.3	4.2 ± 0.3	0.003

Mean ± SD was for variables, P-values are weighted linear regression analysis; RDW: red blood cell distribution width.

### 3.2 Association of RAR and AMD

Table 3 displays the findings of the logistic regression analysis regarding the odds of AMD prevalence. RDW showed a positive correlation with AMD in Model 1 [odds ratio (OR) = 1.1; 95% confidence interval (CI), 1.0–1.2; *p* < 0.05], but it was not statistically significant in Models 2 and 3. Albumin showed a negative correlation with AMD in all models. Model 1 (OR = 1.4; 95% CI, 1.2–1.7; *p* < 0.001), Model 2 (OR = 1.3; 95% CI, 1.1–1.6; *p* < 0.05), and Model 3 (OR = 1.3; 95% CI, 1.0–1.6; *p* < 0.05) showed a significant positive correlation between AMD and RAR. This indicates that each unit rise in RAR corresponded to a 30% increase in the odds of AMD prevalence according to the fully adjusted Model 3. To perform a sensitivity analysis, we further converted RAR from a quantitative variable to a qualitative variable by categorizing it into quintiles. Comparing participants in the Q5 group (highest RAR quintile) to those in the Q1 group (lowest RAR), the odds of AMD prevalence were 1.7 times higher (OR = 1.7; 95% CI, 1.2–2.5; *p* < 0.05). This outcome is also reflected in Models 1 and 2. Therefore, higher RAR values were significantly associated with increased odds of AMD prevalence (*p*-trend < 0.05). Furthermore, we employed multinomial logistic regression to analyze both the early and late AMD. The RAR values showed a significant positive correlation with the odds of early AMD prevalence across the three models. However, this relationship was only observed in the unadjusted model for late AMD, which may be attributed to the limited number of late AMD cases (*N* = 54), leading to insufficient statistical power, as shown in Supplementary Table 2.

**Table 3:** Association between RAR and AMD.

	Model 1 OR (95% CI) P	Model 2 OR (95% CI) P	Model 3 OR (95% CI) P
RDW	1.1 (1.0, 1.2) 0.004	1.1 (1.0, 1.1) 0.129	1.1 (1.0, 1.1) 0.219
Albumin	0.6 (0.4, 0.8) 0.001	0.7 (0.5, 0.9) 0.014	0.7 (0.5, 0.9) 0.023
RAR	1.4 (1.2, 1.7) < 0.001	1.3 (1.1, 1.6) 0.010	1.3 (1.0, 1.6) 0.021
Quintiles of RAR			
Q1(2.3–2.8)	Reference	Reference	Reference
Q2(2.8–3.0)	1.3 (0.9, 1.9) 0.112	1.2 (0.8, 1.7) 0.414	1.2 (0.8, 1.7) 0.394
Q3(3.0–3.1)	1.6 (1.1, 2.3) 0.007	1.3 (0.9, 1.8) 0.243	1.2 (0.8, 1.8) 0.241
Q4(3.1–3.4)	2.0 (1.4, 2.7) < 0.001	1.4 (1.0, 2.0) 0.085	1.4 (1.0, 2.0) 0.089
Q5(3.4–10.2)	2.2 (1.6, 3.0) < 0.001	1.7 (1.2, 2.4) 0.005	1.7 (1.2, 2.5) 0.007
P-trend	<0.001	0.003	0.005

Model 1 adjusted for: none; Model 2 adjusted for: age, gender, and ethnicity; Model 3 additionally included the following variables: smoking status, alcohol consumption, general health condition, diabetes, hypertension, hyperlipidemia, body mass index (BMI), white blood cell (WBC), lymphocyte (LYMP), monocyte (MO), red blood cell (RBC), and platelet (PLT).

### 3.3 Assessment of the linear relationship between RAR and AMD

**Figure 2:**
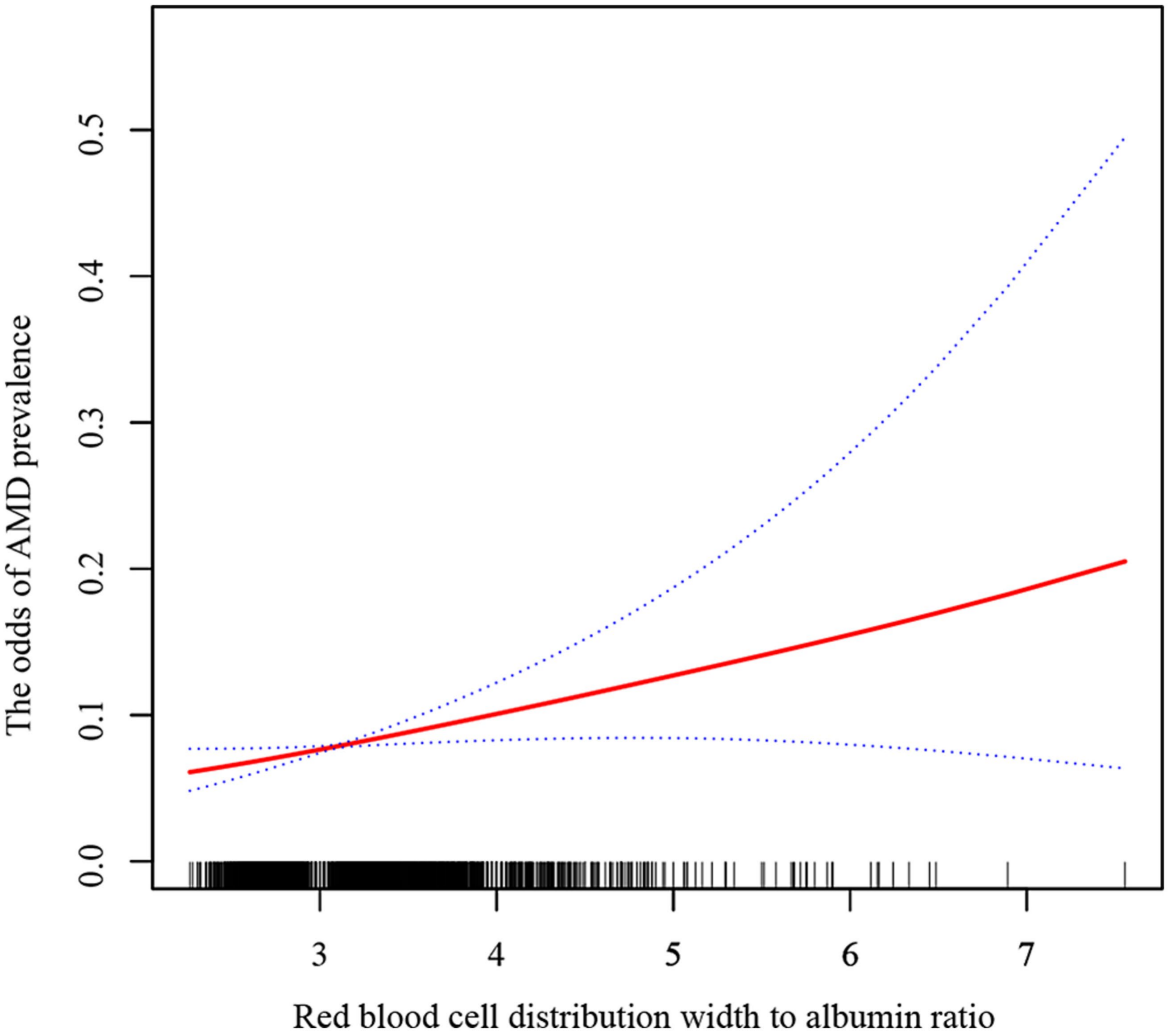
The relationship between RAR and AMD by smoothing curve fitting. The solid red line represents a smooth curve fit between the variables, while the blue lines indicate the 95% confidence interval.

Smooth curve fitting and two-piecewise linear regression models were employed to determine whether there was a linear or non-linear relationship between RAR and AMD based on Model 3 (Figure 2; Table 4). The results showed a log-likelihood ratio greater than 0.05 and a lack of statistical significance at the inflection point (*K* = 3.9). However, the one-line model was found to be statistically significant. Therefore, we can deduce that the one-line model is better suited for illustrating the link between RAR and AMD. The above results suggest a positive and linear correlation between RAR and the odds of AMD prevalence.

**Table 4:** The effect of thresholds on RAR and AMD.

	OR(95% CI) P-value
One-line model	
linear effect	1.3 (1.0, 1.6) 0.021
Two-piecewise linear regression model	
Inflection point(K)	3.9
RAR < K	1.6 (1.1, 2.3) 0.012
RAR > K	1.0 (0.6, 1.6) 0.984
Log likelihood ratio	0.2

### 3.4 Stratification analysis

**Figure 3:**
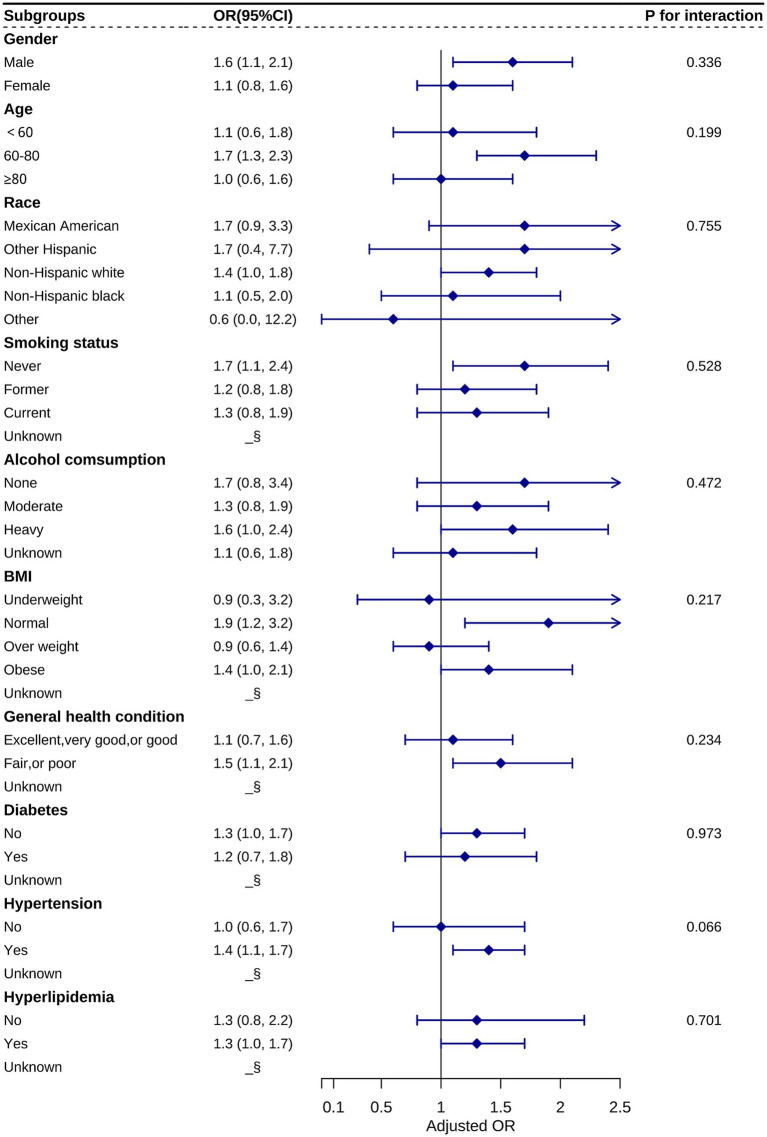
Stratification analysis of the relationship between RAR and AMD based on Model 3. Statistical differences between subgroups could not be observed from the graphs (all P for interactions > 0.05). §: data not shown due to small sample size.

We further stratified the categorical variables among the covariates and analyzed the relationship between RAR and the odds of AMD prevalence in different subgroups based on Model 3. These covariates included gender, age, ethnicity, smoking status, alcohol consumption, BMI, general health status, and cardiovascular disease (Figure 3). Considering the strong correlation between AMD and age, we subdivided age into <60, 60–80, and ≥80 years. After performing the interaction test, no statistically significant differences were observed among the three age subgroups, indicating that the relationship between RAR and the odds of AMD prevalence remained consistent across the three age groups. Meanwhile, the interaction *p*-values were >0.05 for the other categorical variables. These results demonstrate the reliability of the findings based on this regression analysis.

## 4 Discussion

This investigation first established the relationship between RAR and AMD through a nationally representative sample of US adults. Our analysis demonstrates a significant, independent association between elevated RAR and increased odds of AMD prevalence, with this linear positive correlation consistently maintained in multivariate-adjusted models. Our findings highlight the significance of RAR as a novel integrated biomarker of systemic inflammation and nutritional status, providing valuable insights into the role of inflammation in the pathogenesis of AMD.

Inflammation plays a crucial role in the pathological progression of AMD (4). As individuals age, RPE cells undergo functional impairment due to persistent oxidative stress and inflammatory mediators, such as TNF-*α* and IL-6 (29). These mediators activate the complement system and stimulate the release of pro-inflammatory cytokines. Such inflammatory cascades not only exacerbate RPE degeneration but also compromise the blood–retinal barrier and promote choroidal neovascularization, accelerating the progression of AMD (30, 31). Persistent low-grade inflammation, a common characteristic of age-related disorders, highlights the clinical significance of the RAR, a novel biomarker that integrates erythrocyte heterogeneity (as indicated by RDW) (14) and systemic inflammatory-nutritional status (through albumin levels) (19). Elevated levels of RAR are associated with cardiovascular, metabolic, and immune-mediated diseases. This correlation likely arises from mechanisms that involve the amplification of oxidative stress, endothelial dysfunction, and immune-metabolic dysregulation (23–27), positioning RAR as a proxy for systemic inflammatory burden. Our study further demonstrates that elevated RAR levels are significantly associated with increased odds of AMD prevalence (OR = 1.3; 95% CI, 1.0–1.6), reinforcing the association between AMD and systemic inflammation.

RDW is a biomarker that indicates the variability in red blood cell volume and is closely associated with systemic inflammatory states. Inflammatory stimuli, infections, and nutritional or metabolic abnormalities can impact red blood cell production, lifespan, and deformability, resulting in elevated RDW levels (14, 32). In a 2021 case–control study involving 146 participants, Pinna et al. found that RDW levels were significantly higher in patients with RAO than in the control group (15). Elbeyli et al. suggested that RDW is a stronger predictor of central retinal artery occlusion (CRAO) than other inflammatory markers, such as the platelet-to-lymphocyte ratio (PLR), systemic immune-inflammation index (SII), and neutrophil-to-lymphocyte ratio (NLR) (16). Recent reports also indicate that RDW levels are significantly elevated in patients with RVO (17, 18). RDW may serve as a comprehensive indicator of multifactorial disease processes, playing a role in conditions involving oxidative stress, inflammation, and endothelial dysfunction, such as retinal vascular diseases (RAO and RVO). Our study discovered that participants with AMD had significantly higher levels of RDW, as shown in Table 2. However, in multifactorial analyses, RDW revealed a statistically positive association with AMD only in the unadjusted model, reminding us that it may not be used independently as an inflammatory marker for assessing AMD.

Serum albumin is a crucial biomarker for evaluating nutritional status and inflammation. Inflammatory responses enhance capillary permeability, resulting in the leakage of albumin, which ultimately leads to decreased serum albumin levels (20, 33). In a 2019 study, Schultz et al. proposed that the blood–retina barrier (BRB) in non-exudative AMD may display subclinical damage, resulting in the leakage of serum proteins, such as albumin, into the retina. They found significantly elevated levels of albumin expression in the retinas of patients with non-exudative AMD compared to normal controls (34). We hypothesized that patients with AMD experience hypoalbuminemia as a result of inflammatory stimuli. Moreover, our findings show that patients with AMD had lower serum albumin levels, as shown in Table 2. After fully adjusting for covariates, multifactorial regression analysis confirmed the persistent negative correlation between serum albumin and AMD. However, recent studies have demonstrated that RAR exhibits a stronger predictive ability compared to RDW and albumin when considered individually (24, 35). Consequently, our study also emphasizes the combination of these two factors, offering a comprehensive assessment of their impact on the odds of AMD prevalence.

Emerging studies based on the NHANES database validate RAR’s clinical relevance: In 2024, Li et al. found that elevated RAR levels increase the risk of peripheral artery disease in diabetic patients (36). In the same year, Wu et al. demonstrated a correlation between higher RAR and a greater prevalence of kidney stones in the general adult population (37), highlighting the potential of RAR in assessing the risk of metabolic diseases. In 2025, Yu et al. and Shangguan et al. associated elevated RAR with cognitive decline and depression, respectively, further underscoring the role of systemic inflammation and nutritional status in mental health disorders (38, 39). Notably, extending this discovery to ophthalmic diseases, our analysis of 5,370 NHANES participants revealed a positive linear relationship between RAR and the odds of AMD prevalence. Comparing participants in the Q5 group (highest RAR quintiles) to those in the Q1 group (lowest RAR), the odds of AMD prevalence were 1.7 times higher (OR = 1.7; 95%CI, 1.2–2.5), suggesting that RAR may be a quantifiable indicator of retinal inflammatory damage in patients with AMD.

Nevertheless, there are several limitations to this study. First, cross-sectional design limits the ability to establish causality, so future research should incorporate randomized controlled trials or cohort studies. Second, the NHANES database only surveyed the US population, meaning a more extensive survey of a diverse population is required. Third, the study lacks data on the consumption of antioxidants among the participants.

## 5 Conclusion

According to this study, there is a linear positive correlation between RAR and AMD in US adults, which enhances our understanding of the relationship between inflammation levels and the odds of AMD prevalence. Further research is necessary to expand the sample size to validate the correlation between RAR and AMD and to elucidate the impact of RAR on the progression of AMD.

## Data Availability

Publicly available datasets were analyzed in this study. This data can be found at: https://www.cdc.gov/nchs/nhanes/index.htm.
