# Supplementary material for: Red blood cell distribution width-to-albumin ratio and its association with age-related macular degeneration: a population-based cross-sectional study
Source: Front Med (Lausanne). 2025 Apr 15;12:1510756. doi: 10.3389/fmed.2025.1510756 (PMC12037637; doi:10.3389/fmed.2025.1510756)
Supplement: Supplementary file 1 [file Table_1.docx]

***Supplementary tables:***

**Supplementary table1. Summary of AMD status according to RAR quintile grouping**

|  | Total No.(%) | Q1(2.3-2.8) No.(%) | Q2(2.8-3.0) No.(%) | Q3(3.0-3.1) No.(%) | Q4(3.1-3.4) No.(%) | Q5(3.4-10.2) No.(%) |
| --- | --- | --- | --- | --- | --- | --- |
| Without AMD | 4945(93.4) | 1013(95.9) | 1006(94.7) | 955(92.9) | 1000(91.1) | 971(90.5) |
| Early AMD | 371(5.8) | 51(3.8) | 64(4.7) | 72(6.4) | 89(7.6) | 95(7.9) |
| Late AMD | 54(0.8) | 3(0.3) | 8(0.5) | 11(0.7) | 15(1.3) | 17(1.6) |

**Supplementary table2. Multinomial logistic regression model of the relationship with AMD and RAR base on AMD status**

|  | Model 1  OR(95%CI) P | Model 2  OR(95%CI) P | Model 3  OR(95%CI) P |
| --- | --- | --- | --- |
| Without AMD | Reference | Reference | Reference |
| Early AMD | 1.4 (1.1, 1.6) 0.0011 | 1.3 (1.1, 1.6) 0.0151 | 1.3 (1.0, 1.6) 0.0348 |
| Late AMD | 1.6 (1.1, 2.3) 0.0141 | 1.4 (0.8, 2.3) 0.2248 | 1.3 (0.8, 2.3) 0.2971 |

Model 1 adjust for: none;

Model 2 adjust for: age; gender; race;

Model 3 added the following variables to model 2: smoking status, alcohol consumption, general health condition, diabetes, hypertension, hyperlipidemia, body mass index(BMI), White Blood Cell (WBC), Lymphocyte (LYMP),Monocyte (MO), Red Blood Cell (RBC), Platelet (PLT).
